# Network-Based Functional Prediction Augments Genetic Association To Predict Candidate Genes for Histamine Hypersensitivity in Mice

**DOI:** 10.1534/g3.119.400740

**Published:** 2019-10-23

**Authors:** Anna L. Tyler, Abbas Raza, Dimitry N. Krementsov, Laure K. Case, Rui Huang, Runlin Z. Ma, Elizabeth P. Blankenhorn, Cory Teuscher, J. Matthew Mahoney

**Affiliations:** * The Jackson Laboratory, 600 Main St. Bar Harbor, ME, 04609,; † Department of Medicine,; ‡ Department of Biomedical and Health Sciences,; §§ Department of Computer Science,; ‡‡ Department of Neurological Sciences,; †† Department of Pathology and Laboratory Medicine, University of Vermont Larner College of Medicine, Burlington, VT, 05405,; § School of Life Sciences, University of Chinese Academy of Sciences, Beijing 100049, China, and; ** Department of Microbiology and Immunology, Drexel University College of Medicine, Philadelphia, PA,

**Keywords:** Gene prioritization, machine learning, quantitative trait locus, histamine hypersensitivity, Clarkson’s Disease

## Abstract

Genetic mapping is a primary tool of genetics in model organisms; however, many quantitative trait loci (QTL) contain tens or hundreds of positional candidate genes. Prioritizing these genes for validation is often *ad hoc* and biased by previous findings. Here we present a technique for prioritizing positional candidates based on computationally inferred gene function. Our method uses machine learning with functional genomic networks, whose links encode functional associations among genes, to identify network-based signatures of functional association to a trait of interest. We demonstrate the method by functionally ranking positional candidates in a large locus on mouse Chr 6 (45.9 Mb to 127.8 Mb) associated with histamine hypersensitivity (Histh). Histh is characterized by systemic vascular leakage and edema in response to histamine challenge, which can lead to multiple organ failure and death. Although Histh risk is strongly influenced by genetics, little is known about its underlying molecular or genetic causes, due to genetic and physiological complexity of the trait. To dissect this complexity, we ranked genes in the *Histh* locus by predicting functional association with multiple Histh-related processes. We integrated these predictions with new single nucleotide polymorphism (SNP) association data derived from a survey of 23 inbred mouse strains and congenic mapping data. The top-ranked genes included *Cxcl12*, *Ret*, *Cacna1c*, and *Cntn3*, all of which had strong functional associations and were proximal to SNPs segregating with Histh. These results demonstrate the power of network-based computational methods to nominate highly plausible quantitative trait genes even in challenging cases involving large QTL and extreme trait complexity.

Identifying causal variants within quantitative trait loci (QTL) is a central problem of genetics, but genetic linkage often prevents narrowing QTL to less than several megabases (Mb). Thus, QTL may contain hundreds of candidate genes. Instead of revealing the exact gene (or genes) responsible for trait variation, QTL mapping produces positional candidate genes. Rigorously narrowing a QTL by fine mapping with congenic strains can take years or decades, particularly in organisms like mice that have long generation times. Moreover, high-resolution congenic mapping often reveals that the overall QTL effect is due to multiple linked genes within the QTL rather than a single gene (Parker *et al.* 2013; Yazbek *et al.* 2011). Thus, positional data alone are generally insufficient to nominate candidate genes for subsequent biological follow up. To overcome the limitations of mapping data, researchers look within a QTL for plausible candidate genes. However, these selections are typically done by *ad hoc* criteria using prior knowledge or a literature search. This strategy is strongly biased toward prior knowledge and is highly error prone due to missing annotations. There is a need for rigorous and systematic strategies to distinguish among positional candidate genes for mechanistic follow up.

We developed a novel approach to rank positional candidates based on functional association with a trait. To avoid annotation or literature bias, we use functional genomic networks (FGNs), which encode predicted functional associations among all genes in the genome. FGNs such as the Functional Networks of Tissues in Mouse (FNTM) (Goya *et al.* 2015) and HumanBase (Greene *et al.* 2015), are Bayesian integration networks that combine gene co-expression, protein-protein binding data, ontology annotation and other data to predict functional associations among genes. With these networks we can expand on known gene-trait associations to identify genes that were not previously associated with the trait.

Recent studies with functional genomic networks have demonstrated their power to generate novel associations between genes and specific phenotype terms (Guan *et al.* 2010) or biological processes (Ju *et al.* 2013). For example, Guan *et al.* (2010) used a support vector machine (SVM) classifier to identify a gene network associated with bone mineralization. They predicted and validated novel associations between genes and bone mineralization phenotypes for genes that lay outside of all published QTL for bone mineralization phenotypes (Guan *et al.* 2010). Subsequent studies using similar network-based techniques have made novel predictions of hypertension- and autism-associated genes (Greene *et al.* 2015; Krishnan *et al.* 2016). We have expanded these methods to rank genes in a mapped QTL based on multiple putative functional terms and to integrate these rankings with genetic association *p* values from strain surveys. We generated a ranked list for all genes in the QTL that incorporated both the functional and positional scores of each candidate gene.

Our strategy first built trait-associated gene lists from structured biological ontologies (*e.g.*, the Gene Ontology (Ashburner *et al.* 2000; Gene Ontology Consortium 2018) and the Mammalian Phenotype Ontology (Smith and Eppig 2012)) and public transcriptomic data from the Gene expression Omnibus (GEO) (Edgar *et al.* 2002; Barrett *et al.* 2012). We then applied machine learning classifiers to the functional networks of tissues in mice (FNTM) (Goya *et al.* 2015) to identify network-based signatures of the trait-related gene lists. This strategy allowed us to predict gene-trait associations that were not annotated within a structured ontology, overcoming the missing annotation problem.

We applied our approach to a large QTL associated with histamine hypersensitivity (Histh) in mice. Histh in mice is a lethal response to a histamine injection. In insensitive mice, a histamine injection produces an inflammatory response that resolves without further treatment. Mice with the Histh response develop excitation and ear blanching, followed by progressive respiratory distress, vasodilation, anaphylactic shock, and death (Vaz *et al.* 1977; Wang *et al.* 2014). Histh can be induced in a subset of mouse strains by sensitization with Complete Freund’s Adjuvant (CFA). Histh also develops spontaneously in SJL/J animals older than six months of age.

We previously mapped Histh to a locus on Chr 6 (45.9 Mb to 127.8 Mb; the *Histh* locus), which was confirmed using a congenic line (B10.S-*Histh^SJL^*) (Raza *et al.* In Press). Because of the large size of this locus, additional information is required to identify causal variants. To narrow down candidates, we integrated novel genetic association data from interval-specific congenic recombinant lines (ISCRLs) and an inbred strain survey with our network-based functional predictions of Histh-related genes. By augmenting positional data with functional predictions, we dramatically reduced the candidate gene list to a tractable set of high-quality candidates that are implicated in Histh-related processes.

## Materials and Methods

**Figure 1:**
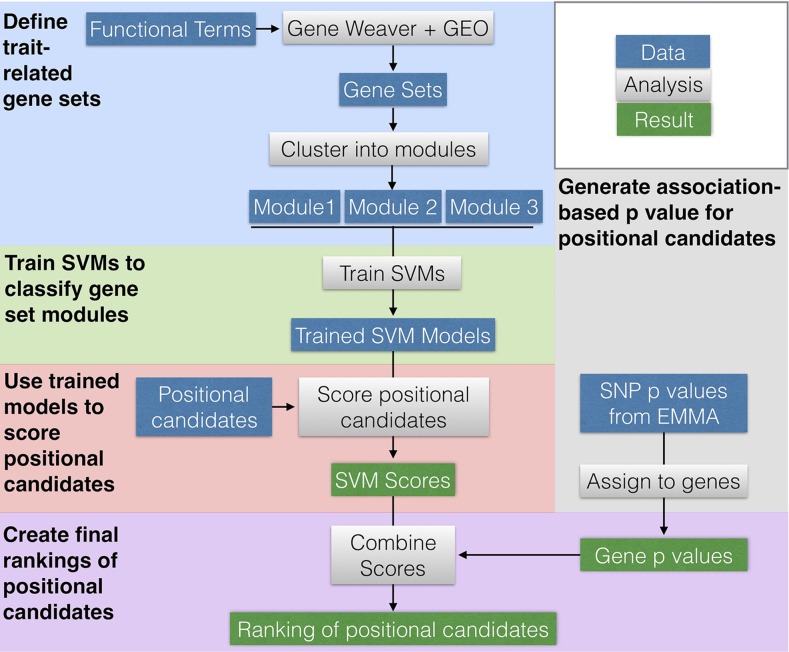
Workflow Overview. The workflow is broken into blocks by color, each with a bolded title. Each block shows how data (blue rectangles) were operated on (gray rectangles) to achieve results (green rectangles). Arrows show the general flow of work and dependence (and independence) of individual analyses.

As a supplement to the computational portion of the methods section, this paper includes an executable workflow (See Data Availability). An outline of the computational workflow is shown in Figure 1. The workflow includes all files and parameters required to recreate the computational portions of this study.

### Animals

A total of 23 mouse strains (129X1/SvJ, A/J, AKR/J, B10.S-*H*2s/SgMcdJ (B10.S), BALB/cJ, BPL/1J, BPN/3J, C3H/HeJ, C57BL/6J, C57BL/10J, CBA/J, CZECHII/EiJ, DBA/1J, DBA/2J, FVB/NJ, JF1/MsJ, MOLF/EiJ, MRL/MpJ, NOD/ShiLtJ, NU/J, PWD/PhJ, PWK/PhJ, SJL/J and SWR/J were purchased from the Jackson Laboratory (Bar Harbor, ME). All mice, including B10.S-*Histh*^*SJL*^ and B10.S-*Histh*^*SJL*^ ISRC lines, were generated and maintained under specific pathogen-free conditions in the vivarium of the Given Medical Building at the University of Vermont according to National Institutes of Health guidelines. All animal studies were approved by the Institutional Animal Care and Use Committee of the University of Vermont.

### Histh Phenotyping

On day 0 mice were injected at two s.q. sites with complete Freund’s adjuvant (CFA) (Sigma-Aldrich, St. Louis, MO) supplemented with 200 *μ*g of *Mycobacterium tuberculosis* H37Ra (Difco Laboratories, Detroit, MI). On D30 histamine hypersensitivity was determined by i.v. injection of histamine (mg/kg dry weight free base) in phosphate buffered saline (PBS). Deaths were recorded at 30 min post injection and the data are reported as the number of animals dead over the number of animals studied. Significance of observed differences was determined by Chi-square with *p*-values <0.05 significant.

### DNA extraction and genotyping

DNA was isolated from mouse tail clippings as previously described (Sudweeks *et al.* 1993). Briefly, individual tail clippings were incubated with 300μL cell lysis buffer (125μ g/mL proteinase K, 100 mM NaCl, 10mM Tris-HCl (pH 8.3), 10 mM EDTA, 100mM KCl, 0.50% SDS) overnight at $55^{\circ }C$. The next day, 150μL of 6M NaCl were added followed by centrifugation for 10 min at $4^{\circ }C$. The supernatant layer was transferred to a fresh tube containing 300μL of isopropanol. After centrifuging for two minutes, the supernatant was discarded, and pellet washed with 70% ethanol. After a final two-minute centrifugation, the supernatant was discarded, and DNA was air dried and resuspended in 50μL TE.

#### Genotyping:

Genotyping was performed using either microsatellite markers in a standard PCR reaction or sequence-specific SNP primers in a phototyping reaction. Polymorphic microsatellites were selected to have a minimum polymorphism of 8bp for optimal identification by agarose gel electrophoresis. Briefly, primers were synthesized by IDT-DNA (Coralville, IA) and diluted to a concentration of 10μM. PCR amplification was performed using Promega GoTaq. The cycling conditions included a two-minute initial denaturation step at 94° followed by 35 cycles of 94° for 30 sec, 55° for 30 sec and 72° for 30 sec followed by a final extension step at 72° for five minutes. Amplicons were subjected to 2% agarose gel electrophoresis and visualized by ethidium bromide and UV light.

#### Phototyping:

Genotyping was performed using sequence-specific primers that differ only at the 3’ nucleotide corresponding to each allele of the identified SNP (Bunce *et al.* 1995). Each primer set was designed using Primer3 to have a Tm of 58-60${}^{\circ }$C, synthesized by IDT-DNA (Coralville, IA), and used at a concentration of 100*μ*M (primer sequences are available in Supplemental File 1). PCR reactions were subjected to multistage (high, medium and low stringency) cycling conditions as described in Supplemental File 2 and if found to be necessary, the cycle conditions at each stage were adjusted to accommodate the optimal annealing temperature. Amplicons were electrophoresed with 10μL Orange G loading buffer on a 1.5% agarose gel stained with ethidium bromide and visualized by UV light. The presence of a SNP-specific allele was scored by observing an amplicon of the expected size in either reaction. Cycling conditions are available in Supplemental File 2.

### Generation of Histh congenic lines and GigaMUGA

B10.S-*Histh^SJL^* ISRC lines were generated by identifying recombinant haplotypes across the *Histh* interval among (B10.S-*Histh^SJL^* × B10.S) × B10.S BC1 mice and then fixed as homozygous lines (Figure 3). To identify potential contaminating background loci segregating among the strains and to further refine the recombination break points of each line, the lines were further genotyped using GigaMUGA arrays (143,259 markers) by the commercial service of Neogen/Geneseek (Lincoln, NE).

### Targeted genetic association testing

We retrieved genotype data (both coding and non-coding) of the 23 mouse strains from the databases at the Sanger Institute (Keane *et al.* 2011) (https://www.sanger.ac.uk/science/data/mouse-genomes-project) and The Jackson Laboratory (Bogue *et al.* 2017) (https://phenome.jax.org/). The lack of representation of wild-derived strains *e.g.*, MOLF/EiJ and others, in these databases was compensated for by genotyping using Illumina Hiseq 2500 platform https://www.illumina.com/. For detailed methods see Supplemental File 3. All these data sources were collated to generate genotype information for a total of 15,302 SNPs across the *Histh* locus (45-128 Mbp, Supplemental File 4).

To calculate associations between genetic polymorphisms and Histh, we used efficient mixed-model association (EMMA) (Kang *et al.* 2008). This method treats genetic relatedness as a random variable in a linear mixed model to account for population structure, thereby reducing false associations between SNPs and the measured trait. To calculate the kinship matrix, we compiled a set of 470,365 SNPs across all strains from the Mouse Genome Informatics database (Mouse Genome Informatics Mouse Genome Informatics Web Site). We removed the SNPs in the congenic interval from the kinship estimation, and generated the kinship matrix with the remaining 455,068 SNPs. We used the likelihood ratio test function (emma.ML.LRT) to generate *p* values. Significance was defined with a Bonferroni correction (p=0.05/15,302). Genomic coordinates included for each SNP using the latest mouse genome build GRCm38.p5/mm10.

### Trait-related gene sets

The positional candidate genes were ranked based on their predicted association with seven functional terms related to the Histh phenotype: “aging”, “*Mycobacterium tuberculosis*”, “cardiac”, “G-protein coupled receptor”, “histamine”, “inflammation”, “type I hypersensitivity”, and “vascular permeability.” We used Gene Weaver (Baker *et al.* 2012) to generate gene sets associated with each term. To do this, we entered each term into the Gene Weaver homepage (https://geneweaver.org). We restricted the search to human, rat, and mouse genes, and to curated lists only. Mouse orthologs for each gene were retrieved using the batch query tool in MGI (http://www.informatics.jax.org/batch_data.shtml). In addition, we used Gene Expression Omnibus (GEO) and PubMed to retrieve expression data sets for each phenotype term. We removed all positional candidates from the gene lists used for training such that no true-positive positional candidates were used for training. The gene lists used are available as a set of zipped text files in Supplemental File 5.

### FNTM network

We trained support vector machines (SVMs) to classify genes in each gene list using features derived from FNTM (Goya *et al.* 2015). In this functional genomic network, genes are nodes, and the edge weights between them are continuous values between 0 and 1 predicting the degree to which each pair of genes is functionally related. Larger values indicate higher predicted functional relatedness. Functional relatedness in this network was predicted through Bayesian integration of data sets from multiple sources, including gene expression, protein-protein interaction data, and annotation to GO terms (Goya *et al.* 2015). We downloaded the top edges of the mouse network on January 15, 2018 from http://fntm.princeton.edu.

### Clustering gene sets

Guan *et al.* (2010) observed that support vector machines trained on 200 to 300 genes yielded the best classification accuracy. Two of our gene lists had fewer than 100 genes. For all lists over 400 genes, we reduced the size of our training sets by clustering each term gene set into modules using the fast greedy (Newman 2004) algorithm in the R/igraph package (Csardi 2006). We applied the fast greedy algorithm iteratively until all modules comprised fewer than 400 genes. Using a maximum modules size of 300 overly fragmented the networks yielding many modules with fewer than 100 genes.

### Machine learning

**Figure 2:**
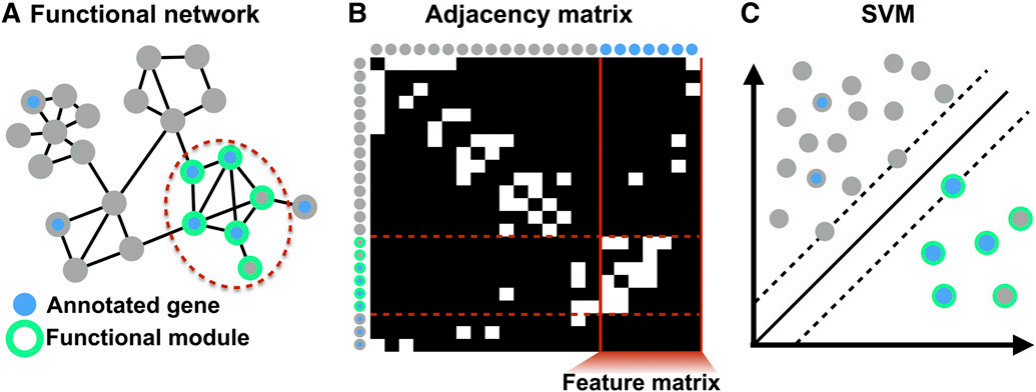
Network-based machine learning for functionally annotating genes. A Known-positive genes annotated to a functional term (blue nodes) are typically densely interconnected in a functional network. B The adjacency matrix of a network is a tabular representation of the connectivity structure of the network in which each row/column corresponds to a node of the network, and connected pairs of nodes have non-zero values in the corresponding cell of the matrix. Note that in general the connections are weighted, but for display we are only showing present/absent links (white/black cells). The connections from every gene in the genome to the known positives form a sub-matrix of the adjacency matrix called the feature matrix (vertical red lines), whose rows are the feature vectors for each gene. C Using the network-based feature vectors for each gene, we train SVMs to distinguish known positives (blue dots) from random genes in the genome (gray dots) to identify the full sub-network corresponding to the true positive genes (green bordered dots and dotted red lines in panels A,B).

To classify positional candidates as belonging to a functional module, we trained SVMs using the connection weights in the FNTM network as features, as described in Guan *et al.* (2010). Briefly, an annotated set of genes (Figure 2A, blue nodes) is used as a set of known positives for the corresponding functional module. Other genes in this module are expected to be strongly functionally connected to these known positives, *i.e.*, have a high probability of functionally interacting with known positives. Each gene, therefore, is represented as a *feature vector* of connection weights to the known positives, which can be visualized as a sub-matrix of the *adjacency matrix* of the network (Figure 2B). Correspondingly, the rows of this matrix are labeled as either known positive or not (Figure 2B, blue dots *vs.* gray dots). We used the e1071 package in R (Meyer *et al.* 2018) to train SVMs to distinguish the known positive genes from an equal-sized set of genes selected at random from outside the known positive list using the network-based feature vectors (Figure 2C). The trained model can then annotate novel genes as belonging to the functional module by classifying any gene in the genome (Figure 2C, green bordered nodes).

We trained 100 SVMs on each module selecting a new set of random genes for each run. We used a linear kernel and 10-fold cross-validation for each SVM. We trained each SVM over a series of cost parameters. We started with the sequence $1\times 10^{-5}$ to $1\times 10^{2}$ by factors of 10, and iteratively narrowed the range of cost parameters until we found a series of eight cost parameters that maximized the accuracy of the SVM (see Workflow).

We calculated the area under the ROC curves (AUC) over all runs in the following way: For a sequence ranging from the minimum SVM score to the maximum SVM score, we quantified all true positives (TP), true negative (TN), false positives (FP) and false negatives (FN). The TP genes in this case were those genes from the known positives that were correctly classified as being in the module (above the SVM score cutoff). TN genes in this case were those genes outside the module that were correctly classified as being outside the module (below the SVM score cutoff). We calculated the AUC across the average curve for all 100 SVMs for each module.

### Positional Candidate Scoring

We used the trained SVMs to score each positional candidate gene in the *Histh* locus. The score for each gene gave an estimate of how functionally related each gene was to each module based on its connection weights to the known module genes in the FNTM mouse network. SVMs classify inputs into two classes, and inputs receive either a positive or a negative score depending on which class the SVM places them. The larger the magnitude of this score, the more confident the classification. Genes with large positive scores were predicted by the SVMs to interact functionally with the genes in the module, while genes with large negative scores were predicted to *not* functionally interact with the module genes. The scale of these SVM scores can vary widely between models. Thus, to compare SVM scores across different trained models, we calculated a false positive rate (FPR) for each gene. The FPR lies between 0 and 1 and can be compared across different models. For each gene’s SVM score we calculated the number of true positives (TP), true negatives (TN), false positives (FP) and false negatives (FN) classified by the SVM. The FPR for a given SVM score was calculated as FP/(FP+TN).

The final functional score for each gene was the $max\left(-log_{10}\left(FPR\right)\right)$ across all modules. This meant that genes with a high functional score for a single module, but low functional scores for other modules, received higher overall scores than genes with moderately high scores across all modules.

### Combined Gene Score

High-quality candidate genes in the locus should not only be functionally related to the trait of interest, but should also segregate with the trait of interest. We thus defined a combined gene score ($S_{cg}$) that combined these two aspects of the analysis:

$$S_{cg}=\frac{-log_{10}\left(p_{EMMA}\right)}{\underset{pos.cand.}{\text{max}}-log_{10}(p_{EMMA}))}+\frac{-log_{10}\left(FPR_{SVM}\right)}{\underset{pos.cand.}{\text{max}}-log_{10}\left(FPR_{SVM}\right)},$$

where the denominators of the two terms on the right hand side are the maximum values of $-log_{10}\left(p_{EMMA}\right)$ and $-log_{10}\left(FPR_{SVM}\right)$ over all positional candidates in *Histh*, respectively, which normalizes the functional and positional scores to be comparable to each other. EMMA *p* values for SNPs were assigned to the nearest gene within 1 megabase using the R package biomaRt (Durinck *et al.* 2005, 2009) (Supplemental Table 1). Genes for which more than one SNP was assigned were given the maximum $-log_{10}\left(p_{EMMA}\right)$ across all SNPs associated with that gene. The full matrix of combined scores across all gene sets is in Supplemental Table 2. The rows of this matrix are sorted by the maximum gene score across all gene lists.

### Data availability

A reproducible workflow in R markdown is available on GitHub (https://github.com/MahoneyLab/HhsFunctionalRankings). This workflow contains all code required to reproduce the figures and results presented in this manuscript.

The data used as input for the workflow, as well as intermediate and final results, are available on figshare (https://figshare.com/articles/Data_required_to_run_HhsFunctionalRankings_workflow/8205356). The data set is called under HhsFunctionalRankings. Supplemental material available at figshare: https://doi.org/10.25387/g3.9989117.

## Results

### Generation of interval specific recombinant congenic lines (ISRCL) across the Histh locus

**Figure 3:**
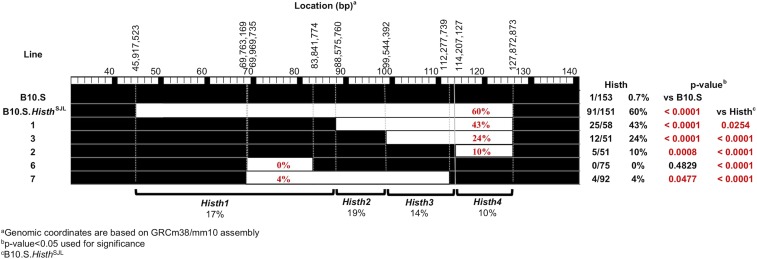
Interval specific recombinant congenic line (ISRCL) mapping identified four linked QTL controlling Histh. ISRCLs were injected (D0) with CFA and subsequently challenged (D30) by i.v. injection of 75 mg/kg histamine to determine histamine hypersensitivity. Deaths were recorded at 30 min post injection and the data are reported as the number of animals dead over the number of animals studied. Significance of observed differences was determined by a $\chi^{2}$ test with *p*-values ≤0.05 considered significant.

In prior work, we mapped the genetic locus regulating susceptibility to age- and/or inflammation (CFA)-dependent sensitivity to histamine on Chr 6 in SJL/J mice (Raza *et al.* In Press). The B10.S-*Histh^SJL^* congenic mice exhibited Histh and carry a large ≈83 Mb region of SJL/J between 45.9 Mb to 127.8 Mb on the resistant B10.S background (Raza *et al.* In Press) (MGI:6360897). This large QTL includes 628 protein coding genes. To narrow this region, we generated five ISRCLs using B10.S-*Histh^SJL^* x B10.S backcross mice and assessed their susceptibility to Histh (Figure 3). Under an additive model, these data suggest that *Histh* is composed of four sub-QTL which we have designated *Histh1* (MGI: 6362992), *Histh2* (MGI: 6362994), *Histh3* (MGI: 6362996), and *Histh4* (MGI: 6362997), each contributing 17%, 19%, 14% and 10%, respectively, to the overall penetrance. Importantly, for each sub-QTL this makes positional candidate gene identification using interactive high-resolution congenic mapping impractical.

### Inbred strain survey of Histh

To investigate whether the Histh phenotype is unique to SJL, we assessed histamine responses for 23 inbred mouse strains (including SJL/J and B10.S; Table 1). These strains were chosen using haplotype structure across the Histh interval to identify additional mouse strains that are likely to share a susceptible *Histh* allele (data not shown). 129X1/SvJ, ALR/LtJ, BPN/3J, FVB/NJ, NOD/ShiLtJ, NU/J, SJL/BmJ and SWR/J mice were identified as having similar haplotype structure as SJL/J at the *Histh* locus. ALR/LtJ and SJL/BmJ mice required embryo recovery and were therefore not included. Histh phenotyping identified FVB/NJ, SWR/J, and NU/J mice as Histh-susceptible, whereas 129X1/SvJ, NOD/ShiLtJ, and BPN/3J were resistant. Taken together with our earlier data, these results indicate that Histh susceptibility segregates among a unique subset of SJL/J-related strains (Petkov *et al.* 2004).

**Table 1:** A survey Histh phenotypes across 23 inbred mouse strains

Strain	HA	Strain	HA	Strain	HA
A/J	0/8	CZECHII/EiJ	0/8	**NOD/ShiLtJ**	**0/8**
AKR/J	0/8	DBA/1J	0/8	**129X1/SvJ**	**0/8**
BALB/cJ	0/8	DBA/2J	0/8	**BPN/3J**	**0/8**
BPL/1J	0/8	JF1/Ms	0/8		
C3H/HeJ	0/8	MOLF/EiJ	0/8	**FVB/NJ**	**6/8**
C57BL/10J	0/8	MRL/MpJ	0/8	**NU/J**	**5/8**
C57BL/6J	0/7	PWD/PhJ	0/12	**SJL/J**	**12/12**
CBA/J	0/8	PWK/PhJ	0/6	**SWR/J**	**6/8**

Cohorts of CFA injected 8- to 10-week old mice were challenge 30 days later with 75 mg/kg HA by i.v. injection, and deaths recorded at 30 min. Results are expressed as the (number of animals dead)/(number of animals studied). The last column contains strains with haplotype structure similar to SJL/J in bold typeface. These strains are divided into those that did not develop Histh (top), and those that did (bottom).

### Targeted genetic association analysis for Histh

Our result from previous linkage analysis (Raza *et al.* In Press) and congenic mapping localized *Histh* to an ≈83 Mb region on Chr 6 between 45.9 Mb to 127.8 Mb. Given that Histh-susceptibility is restricted to a unique subset of inbred strains, particularly the closely related SJL/J, FVB/NJ, and SWR/J, we performed a targeted association analysis between SNPs in the *Histh* locus across all 23 inbred strains (*cf*. Benson *et al.* (2017)).

**Figure 4:**
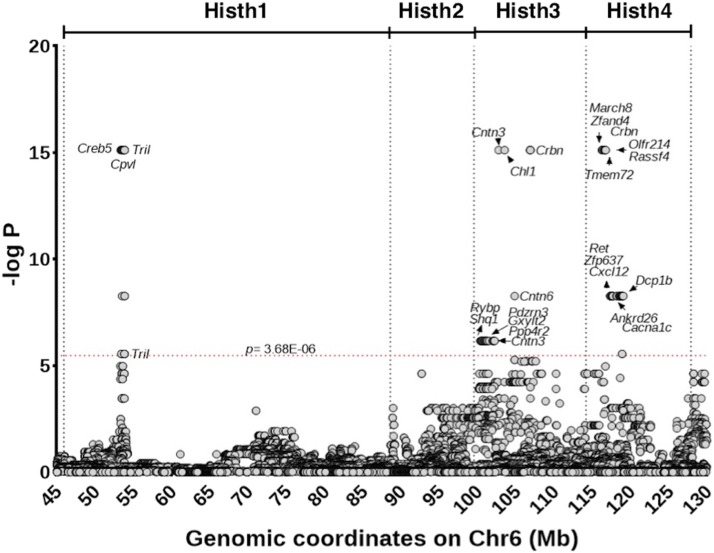
Targeted genetic association analysis for Histh. Negative log-transformed *p* values of SNP associations with Histh. Genomic coordinates (mm10 Mbp) of each SNP are shown along the *x*-axis. Each circle denotes a single SNP. Gene names are included for SNPs that crossed *p*-value threshold of $3.68\times 10^{-6}$ shown with a red dotted line. The location of Histh sub-QTL are shown at the top of the figure.

We tested the association of 15,302 SNPs across the *Histh* locus using efficient mixed-model association (EMMA) (Kang *et al.* 2008). A total of 84 SNPs in 23 genes showed significant associations ($p\leq 3.68\times 10^{-6}$) (Figure 4, Supplemental Table 3). The majority of the significant hits were intronic (71%), non-coding (12%), intergenic (4%) or regulatory (5%) variants. Interestingly, there was overlap between three of the four *Histh* sub-QTL (Figure 3) and SNP-association peaks.

### Network-based prediction of Histh-associated genes

To predict functional candidates among the positional candidates in the *Histh* locus, we delineated a list of Histh-associated biological processes and trained machine learning classifiers to identify sub-networks of functional genomic networks associated with each of these processes. An overview of our workflow is in Figure 1. We first defined gene sets that were related to seven terms that are functionally related to the Histh phenotype.

The terms and their justifications are as follows:

- *Type I hypersensitivity/Anaphylaxis*: The death response following systemic histamine challenge exhibits symptoms of type I hypersensitivity/anaphylaxis including respiratory distress, vasodilation, and anaphylactic shock (Vaz *et al.* 1977).
- *Cardiac*: There is evidence suggesting that anaphylactic shock in mice is associated with decreased cardiac output, rather than solely a function of systemic vasodilation (Wang *et al.* 2014).
- *Histamine*: Histh is elicited by a systemic histamine challenge (Raza *et al.* In Press).
- *G-protein coupled receptor*: Histamine receptor H_1_ (*Hrh1*) signaling is required for the Histh phenotype, and all histamine receptors belong to the family of G-protein coupled receptors (Hill *et al.* 1997).
- *Aging*: Spontaneous Histh develops after six months of age in sensitive mouse strains (Raza *et al.* In Press).
- *Inflammation*: Treatment with pro-inflammatory CFA induces Histh in sensitive mouse strains.
- *Tuberculosis*: Histh is induced in some mouse strains by CFA, which contains inactivated *Mycobacterium tuberculosis* (Raza *et al.* In Press).
- *Vascular permeability*: The Histh response includes vascular leakage in skin and skeletal muscles as assessed by Miles’assay (Raza *et al.* In Press).

We used Gene Weaver, the Gene Expression Omnibus (GEO), and PubMed to retrieve gene sets associated with each of these terms (see Materials and Methods). The gene sets ranged in size from 651 to 1466 genes. Because Guan *et al.* (2010) found that SVMs trained on gene sets with around 300 genes performed best for network-based functional prediction, we clustered large gene sets into modules of approximately 300 genes and analyzed each module separately (see Materials and Methods). Supplemental Table 4 shows the number of genes in each module, as well as the top five enrichment terms for each using the R package gProfileR (Reimand *et al.* 2018). Multiple members of these gene sets are encoded in the *Histh* locus. For example, *Hrh1* was a member of the Anaphylaxis gene set. To reduce bias in classification, we removed all such genes from each gene set before SVM training. We then trained an ensemble of 100 SVMs on each module gene set. We calculated ROC curves for each model to quantify the ability of each set of SVMs to distinguish genes inside the module gene set from all genes outside the module gene set. AUCs ranged from 0.9 to 0.975 indicating that the SVMs were able to classify the genes in each list robustly. In other words, each gene set used to define a putative Histh-related process forms a distinct subnetwork of the full functional genomic network. We then applied the trained SVM models to the positional candidate genes in the *Histh* locus. By classifying each positional candidate, we can identify genes that are likely to be functionally associated with each module gene set. For example, for the Anaphylaxis module gene set, the histamine receptor *Hrh1* received a positive score indicating that the SVMs predicted that it belonged to the Anaphylaxis gene set despite its absence from the training set. This example provides a positive control and shows that the SVMs identify biologically relevant patterns in the functional genomic network. In addition to the SVM score, we calculated a false positive rate (FPR) for each gene (see Materials and Methods). Low FPRs indicate high confidence in the classification. The details of this analysis are described in an executable workflow as a companion to this paper (see Data Availability).

### Integration of functional enrichment with genetic association

**Figure 5:**
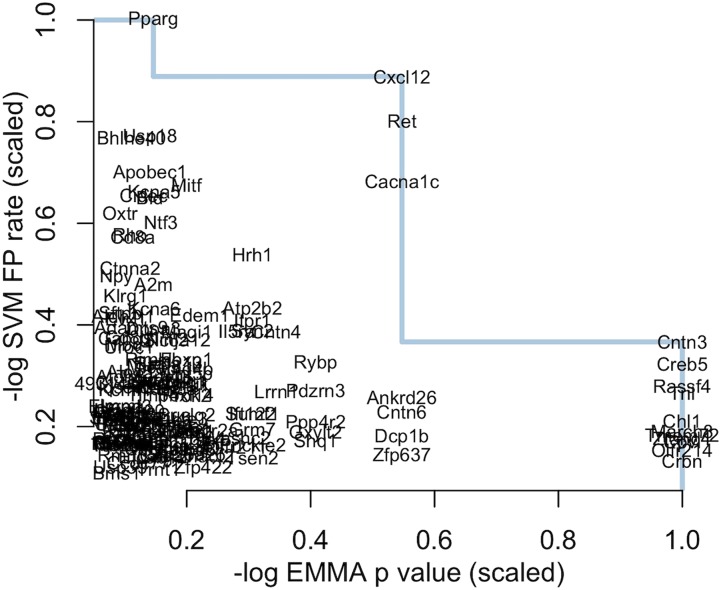
Two axes of gene scoring. Gene names are plotted by their $-log_{10}\left(p_{EMMA}\right)$ on the *x*-axis and the $-log_{10}\left(FPR_{SVM}\right)$ on the *y*-axis. Both scores were scaled by their maximum value for better comparison. Genes farther to the right were associated with SNPs that segregated with Histh. Genes higher up on the *y*-axis have stronger functional association with gene modules. The blue line marks the Pareto front. Genes on this line maximize the two scores and are the best candidates based on the combination of both scores.

Genes that are predicted to be highly functionally related to the trait may not have functionally variant alleles in the study population, and may therefore be unlikely to drive the observed strain differences in Histh. To identify genes that were likely to have functionally relevant polymorphisms, we integrated functional scores with SNP association *p* values to focus only on those candidates that satisfied both criteria. By plotting the maximum functional score for a gene, $-log_{10}\left(FPR_{SVM}\right),$ *vs.* the $-log_{10}\left(p_{EMMA}\right)$ (normalized to the max values; see Materials and Methods), we can identify genes that were predicted to be both highly functionally related to Histh phenotype and likely to have functional polymorphisms that segregated with Histh susceptibility (Figure 5). The blue line in Figure 5 traces along the Pareto front of the gene set in this space. For any gene on this line, finding a gene with a stronger functional association means finding a gene with lower SNP *p* value, and *vice versa*. The genes near the Pareto front have either segregating polymorphisms or are predicted to be functionally related to Histh, or both. All such genes are potentially good candidates for experimental follow-up.

**Figure 6:**
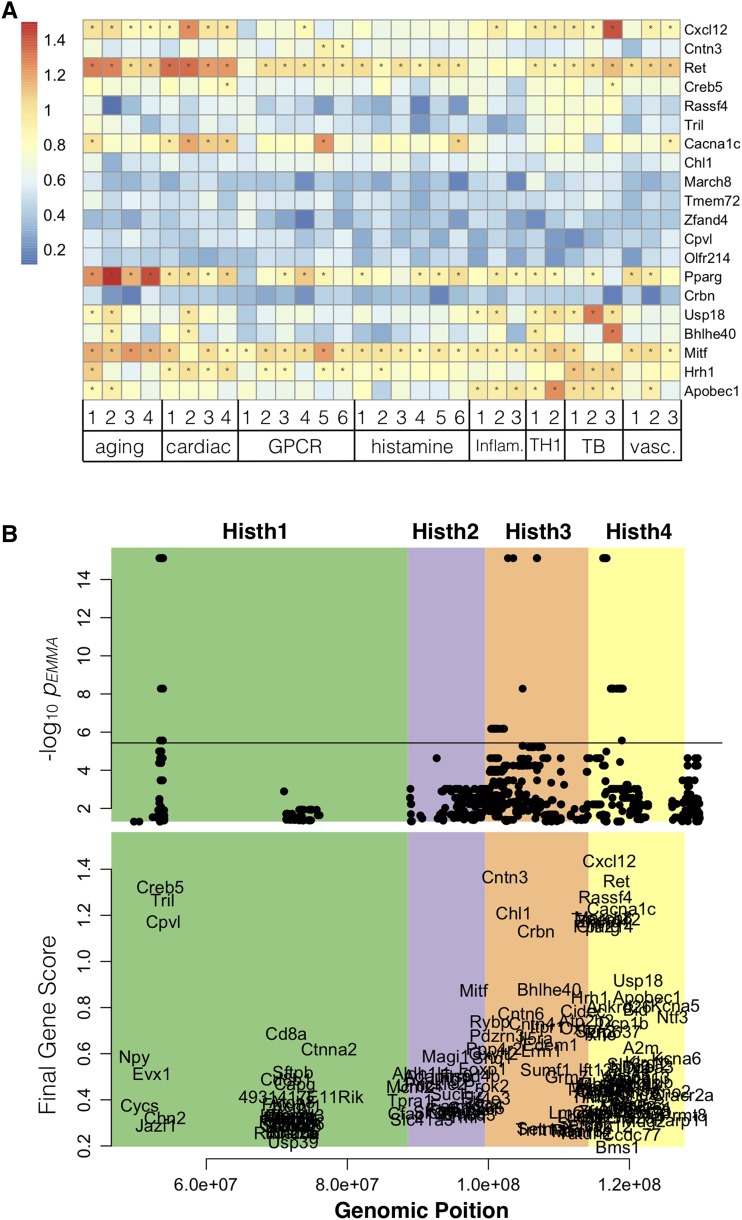
Final gene scores. Gene functional values were combined with SNP associations to assign each gene a final gene score ($S_{cg}$). Higher gene scores indicate better candidates. A Heat map showing the final score of each of the top 20 ranked genes for each gene module. To aid visualization of the strongest candidates, asterisks in each cell indicate where candidate genes were associated with a module with an $FPR_{SVM}\leq 0.2$. B The top panel shows individual SNPs plotted at their genomic location (*x*-axis) and their ${-\text{log}}_{10}\left({\text{p}}_{\text{EMMA}}\right)$ (*y*-axis). All SNPs with nominally significant *p* value (p≤0.05) are plotted. The horizontal line indicates the Bonferroni corrected significance cutoff (p≤0.05/13598). The four sub-QTL are demarcated by background color and are labeled at the top of the figure. The bottom panel shows genes plotted at their genomic location (*x*-axis) and their final gene score ($S_{cg}$) (*y*-axis) to demonstrate how the final ranked genes align with the SNP association data.

To rank the candidates with a single score, we defined a final gene score ($S_{cg}$) for each gene, which is the sum of the (normalized) $-log_{10}\left(FPR\right)$ and the $-log_{10}\left(p_{EMMA}\right)$ (Figure 6). This score prioritizes candidates in the upper right quadrant with simultaneously high positional and functional scores. The genes in the upper right quadrant—*Cxcl12*, *Ret* and *Cacna1c*—had near-maximal scores along both axes and were therefore ranked as the best candidates for follow-up. The full table of gene scores by module can be seen in Supplemental Table 2.

In addition to identifying the top-ranked gene over the full *Histh* locus, we identified a top-ranked gene for each sub-QTL identified through congenic mapping. Figure 6A shows the functional associations across all modules of the top 20 genes ordered by final gene score ($S_{cg}$). The full matrix of scores for all ranked genes can be found in Supplemental Table 2.

## Discussion

In this analysis, we identified a small set of positional candidate genes in a large locus by combining SNP associations with predicted functional associations to Histh. SNP association tests resulted in groups of SNPs with many significant and indistinguishable *p* values. Figure 5 shows a group of genes in the lower right corner that have maximally significant *p* values. These genes are indistinguishable by *p* value alone, but the functional score in this pipeline differentiated these genes based on relatedness to the processes of interest. Furthermore, multiple genes that were highly ranked by the full pipeline did not receive maximally significant *p* values from the SNP association tests. We assigned SNPs to genes based on proximity, but this assignment can produce false associations between genes and phenotypes. For example, a SNP could tag a regulatory variant that influences multiple nearby genes, only some of which are associated to the trait. Moreover, the SNP may not completely segregate with the causal variant. Likewise, the complexities of genetic architecture, including epistasis, may result in reduced significance for univariate SNP associations for truly causal SNPs. Thus, genes with less than optimal *p* values may be overlooked if *p* value were the only ranking criterion. By adding a functional criterion to the ranking process, we can buffer against mapping complexities and gene assignment errors and identify which of the positional candidates are also likely to be functionally related to Histh. The final list of genes was highly plausible and can be followed up relatively easily with modern genetic editing techniques.

### High-quality candidates for Histh

Three genes in the final ranked list deserve particular attention: *Cxcl12*, *Ret*, and *Cacna1c*. These genes did not have the most significant *p* values across the locus, but were predicted to be highly functionally related to Histh-related processes (Figure 5). The top-ranked gene, *Cxcl12* (also known as stromal cell-derived factor 1 (Sdf1)), is chemotactic for mast cells via the chemokine receptor *Cxcr4* (Ghannadan *et al.* 2002). Mast cells are major drivers of pathological events in anaphylaxis (Lieberman and Garvey 2016), demonstrating that the final predictions are highly relevant to Histh. Additionally, CXCL12/SDF1 secreted by tumor cells is associated with increased endothelial permeability, both locally and systemically. The second-ranked gene *Ret* encodes the ret proto-oncogene, a cellular tyrosine kinase transmembrane receptor, the activation of which stimulates multiple downstream pathways involved in cell differentiation, growth, migration, and survival, inflammation (Rusmini *et al.* 2013) and the development of the cardiovascular system (Hiltunen *et al.* 2000). Alleles of this gene could conceivably modify multiple processes underlying Histh, including the both the anatomical background susceptible to Histh and the acute response to histamine. *Ret* was significantly associated with multiple functional gene sets (Figure 6A). The third-ranked gene, *Cacna1c*, calcium channel, voltage-dependent, L type, alpha 1C subunit, which is expressed in the heart, blood vessels, and central nervous system (Mouse Genome Informatics Mouse Genome Informatics Web Site). Mutations in *Cacna1c* are associated with electrophysiological alterations in the heart (Napolitano *et al.* 2015; Hedley *et al.* 2009) suggesting a possible role for *Cacna1c* in impaired cardiac function in Histh. Interestingly, SNPs in human *CACNA1C* were recently associated with chronic spontaneous urticaria (*i.e.*, spontaneous episodes of hives and/or angioedema) and antihistamine drug response (Yan *et al.* 2018). These results suggest a direct connection between *Cacna1c* and the histamine response.

All of the above genes lie in the *Histh4* locus, which accounts for only a portion of the total variation in the Histh phenotype. In the *Histh3* locus, the highest-ranked candidate gene was *Cntn3*, which encodes for contactin 3, also called brain-derived immunoglobulin superfamily protein 1 (BIG-1) or plasmacytoma-associated neuronal glycoprotein (PANG), was first reported in 1994 in rats (Connelly *et al.,* 1994; Yoshihara *et al.,* 1994). Genetic variants of human *CNTN3* are associated with an autism spectrum disorder (Morrow *et al.,* 2008; Hussman *et al.,* 2011) and vascular abnormalities underlying abdominal aortic aneurysms (Elmore *et al.* 2009). The latter suggests a potential connection to impaired cardiac function during histamine challenge (Elmore *et al.* 2009). Intriguingly, *CNTN3* is near a segregating SNP for Systemic Capillary Leak Syndrome (SCLS) from a human GWAS. SCLS is an extremely rare disease characterized by transient but potentially lethal episodes of diffuse vascular leakage of proteins and fluids into peripheral tissues, resulting in massive whole-body edema and hypotensive shock. The pathological mechanisms and genetic basis for SCLS remain elusive (Xie *et al.* 2013), but SCLS shares many phenotypic properties with Histh in mice. In particular, SCLS attacks are diagnosed based on the clinical triad of hypotension, elevated hematocrit, and hypoalbuminemia, all of which naturally occur in the Histh-sensitive SJL mouse strain (Raza *et al.* In Press). The potential association between *CNTN3* and SCLS, therefore, lends credence to its possible functional role in Histh as well. Indeed, *CNTN3* was not only a positional candidate in the SCLS GWAS, but was contained within functional terms that were enriched among the top positional candidate genes (Xie *et al.* 2013), indicating that CNTN3 functions in concert with other genetic risk factors for SCLS.

In the *Histh1* locus, the top hits in were *Creb5* and *Tril*. *Creb5* codes for cAMP responsive element binding protein 5. *Creb5* has high expression in the heart (Fagerberg *et al.* 2014) and has been implicated in cardiac function and pathology (Schisler *et al.* 2015). Additionally, CREB5 is as component of the age-associated inflammatory network underlying disregulated cytokine expression, i.e., inflammaging. *Tril* is Tlr4 interactor with leucine-rich repeats and is a functional component of Tlr4 complex involved with LPS signaling and is highly expressed in the kidney (Carpenter *et al.* 2009), indicating a potential role for *Tril* in blood pressure regulation. *Tril*(−/−) mice also produce lower levels of multiple proinflammatory cytokines and chemokines within the brain after *E. coli* and LPS challenge (Wochal *et al.* 2014) and is required for Tlr3 signaling, suggesting a potential role in immune modulation. There were no significant hits in the *Histh2* locus.

None of the prioritized genes here have been tested specifically for association with Histh, and further experimental validation will be required. However, the above genes each have compelling functional associations that can inform follow-up studies.

### Computation and quantitative trait gene prediction

Definitive functional validation of a quantitative trait gene (QTG) has traditionally required either congenic mapping to resolve an extremely narrow QTL, or *ad hoc* nomination of a candidate gene for direct experimentation. The advent of modern genetic technologies, such as CRISPR/Cas9 (Hsu *et al.* 2014), allow relatively fast and inexpensive allelic manipulations, so the burden of QTG prediction is moving toward a regime in which a small handful of strong candidates can be followed up individually. Importantly, many QTL, including *Histh*, contain multiple causal variants, so fine-mapping alone cannot provide definitive validation. Therefore, computational tools that can identify a small number of reasonable candidates can be a significant aid in biological follow-up. We have presented an integrative strategy for ranking genes in a QTL by combining predicted functional associations to the trait with SNP associations. Our method produces a full ranked list of genes in the locus providing researchers with the potential to validate multiple targets. To this end, the *Histh* QTL represents an extreme use case for QTG prediction–a large, polygenic QTL associated with a physiologically complex trait.

One major limitation to our approach is the decision of which functional terms to include for network-based prediction. The better tailored this set is to the trait of interest, the greater confidence we can have in the final predictions. In principle, the inclusion of a spurious functional term could skew the rankings toward genes that are functionally associated with the spurious term but irrelevant to the trait of interest. One potential way around this issue is to use functional data, such as transcriptomics, directly from the mapping population. However, in some cases, including Histh, the relevant tissue in which to measure gene expression may not be obvious. Alternatively, one could consider distinct rankings for each functional term. In any case, the researcher will have to exercise some measure of judgment in the prioritization process. However, by transferring the judgments from a large list of positional candidate genes to a smaller and more tractable list of trait-related biological processes, we have shown that we can arrive at a strong set of follow up candidates that would have evaded naive *p* value filters and are relatively unbiased by findings published in the literature.

Another limitation to this approach is our method of assigning *p* values to genes. We currently use the *p* value from the single best SNP within 1Mb of the gene body, and these SNPs may or may not be tagging the gene they are assigned to. There exist multiple methods for assigning gene-based *p* values based on multiple SNP *p* values, such as the Versatile Gene-Based Test for Genome-wide Association Studies (VEGAS) (Liu *et al.* 2010), Combined Association Test for Genes (COMBAT) (Wang *et al.* 2017), and eigenMT (Davis *et al.* 2016). However, none of these methods are directly applicable to the current experiment, and deriving a gene-based test statistic that is applicable here is beyond the scope of this study. We will be investigating this possibility for future implementations of this method.

The final output of our method, a ranked list of positional candidate genes, is easy to interpret, and provides researchers with a clear set of hypotheses to test in the lab. While this approach cannot definitively identify the causal gene or genes in a locus, it does provide a much-reduced set of plausible candidates to test.
